# Chronic Wasting Disease in Farmed Cervids, South Korea, 2001–2024

**DOI:** 10.3201/eid3204.251046

**Published:** 2026-04

**Authors:** Young Pyo Choi, Yu-Ran Lee, Hoo Chang Park, Yoon Hee Lee, Gordon Mitchell, In-Soon Roh, Hyun-Joo Sohn

**Affiliations:** Korea Brain Research Institute, Daegu, South Korea (Y.P. Choi); World Organisation for Animal Health Reference Laboratory for Chronic Wasting Disease, Gimcheon, South Korea (Y.-R. Lee, H.C. Park, Y.H. Lee, I.-S. Roh, H.-J. Sohn); National and World Organisation for Animal Health Reference Laboratory for Scrapie and Chronic Wasting Disease, Canadian Food Inspection Agency, Ottawa, Ontario, Canada (G. Mitchell)

**Keywords:** Chronic wasting disease, CWD, prions and related diseases, farmed cervid, elk, deer, prevalence, South Korea

## Abstract

Chronic wasting disease (CWD) was identified in imported elk in South Korea in 2001 and has spread among cervids nationwide. The country’s surveillance and control policy culls cervids from any CWD-positive farms, and prevalence during 2020–2024 was <0.5%. Maintaining low prevalence in cervids will limit livestock, wildlife, and human CWD exposure.

Chronic wasting disease (CWD) is a highly contagious prion disease in free-ranging and farmed cervids, affecting species such as sika deer (*Cervus nippon*), red deer (*C. elaphus*), and elk (*C. canadensis*). Since it was first recognized in Colorado, USA, in the 1960s (*1*), CWD has spread widely across North America, where cases have been identified in 36 US states and 5 provinces of Canada (*2*,*3*). CWD has also been detected in parts of the Scandinavian Peninsula (*4*,*5*). In South Korea, CWD was first detected in 2001 in elk imported from Canada, and cervid cases occurred annually during 2016–2024 (*6*–*9*). To study of the epidemiology of CWD in cervids and understand the exposure risk other species, we assessed CWD occurrence among farmed cervids in South Korea during 2001–2024.

## The Study

South Korea conducts CWD surveillance in accordance with the Korean Act on the Prevention of Contagious Animal Diseases, under which CWD is designated as a Type 2 infectious disease (Appendix). The World Organisation for Animal Health Reference Laboratory at the Animal and Plant Quarantine Agency in South Korea performs CWD diagnosis for surveillance. The Korea Animal Health Integrated System maintains detailed CWD occurrence records (https://home.kahis.go.kr). We obtained annual farmed cervid population data from Ministry of Agriculture, Food and Rural Affairs of Korea reports.

CWD surveillance in farmed cervids in South Korea comprises 2 primary categories: high-risk and routine slaughter. High-risk animals include cervids found dead or exhibiting clinical signs suggestive of CWD, animals culled from CWD-positive farms, and animals culled from farms epidemiologically linked to CWD-positive farms. When CWD is confirmed in a high-risk animal, all remaining animals on the affected and epidemiologically linked farms are culled, as detailed elsewhere (*10*). For routine slaughter surveillance, brain and lymph node samples from cervids slaughtered for human consumption are routinely collected and sent for testing. 

Since 2001, a total of 429 farmed cervids in South Korea have tested positive for CWD (Table 1; Appendix Table 1). During 2001–2005, all CWD-positive animals were elk imported from Canada. After a 5-year period with no cases, CWD reemerged in 2010, affecting elk, red deer, and sika deer. The 19 cases reported in 2010, comprising 6 elk, 6 red deer, and 7 sika deer, were the first direct evidence of domestic CWD transmission. After a second 5-year interval (2011–2015), CWD reemerged in 2016, with 44 infected animals across the 3 cervid species at 8 farms. Since 2016, CWD has been detected nearly annually at a rate of 13–104 cases per year across multiple farms, indicating its endemic status in South Korea’s farmed cervids. Of note, cases in red and sika deer have sharply declined, and fewer annual cases were reported during 2020–2024.

**Table 1:** Occurrence stage and animals affected by CWD in farmed cervids, South Korea, 2001–2024*

Occurrence stage	No. farms	CWD testing, no. positive/no. tested			
		Total	Red deer (*Cervus elephus*)	Elk (*C. canadensis*)	Sika deer (*C. nippon*)
Imported elk stage, 2001–2005					
2001	4	9/177	–	9/177	–
2004	5	12/75	–	12/75	–
2005	1	2/130	–	2/130	–
Initial domestic transmission stage, 2010	3	19/185	6/80	6/82	7/23
Endemic stage, 2016–2024					
2016	8	44/299	22/62	6/151	16/86
2018	6†	13/359	7/91	5/218	1/50
2019	4‡	62/391	52/162	10/224	0/5
2020	4	104/672	9/65	70/471	25/136
2021	3†	21/290	4/46	14/171	3/73
2022	7†	60/463	1/12	53/367	6/84
2023	5	34/573	–	34/481	0/92
2024	3§	49/439	0/5	46/429	3/5
Total	53	429/4,053	101/523	267/2,976	61/554

*CWD, chronic wasting disease; –, no species testing. †Includes 1 recurrence farm in Gyeongnam province where CWD recurred after remediation and subsequent reintroduction of cervids. ‡Includes 1 recurrence farm in Chungnam province where CWD recurred after remediation and subsequent reintroduction of cervids. §Includes 1 recurrence farm in Jeonnam province where CWD recurred after remediation and subsequent reintroduction of cervids.

Of the 429 CWD cases in farmed cervids during 2001–2024, most (97.9%) were detected through high-risk surveillance, and only 9 (2.1%) were identified through routine slaughter surveillance. The percentage of CWD cases detected through high-risk surveillance varied by occurrence stage: 78.3% of cases were detected among high-risk animals during the imported elk stage (2001–2005), 89.5% during the initial domestic transmission stage (2010), and 99.5% during the endemic stage (2016–2024). Although most (71.7%, 276/385) cases during the endemic stage were from animals culled on CWD-confirmed farms within the high-risk category, cases during the imported elk stage were more evenly distributed across the 3 high-risk groups. Among the high-risk groups, animals found dead or exhibiting clinical signs had the highest CWD positivity rates during the endemic (36.7%) and imported elk (9.5%) stages, whereas animals culled from CWD-confirmed farms showed the highest positivity rate (23.5%) during the initial domestic transmission stage. 

Since 2014, more than 2,800 wild cervids have been tested for CWD. Surveillance mainly targeted Korean water deer (*Hydropotes inermis argyropus*; 85.4% of tested animals) on the mainland and Siberian roe deer (*Capreolus pygargus*) on Jeju Island, but no positive cases had been detected by 2024 (Table 2).

**Table 2:** Surveillance results for CWD in farmed cervids, South Korea, 2001–2024*

Stage†	Farmed cervids									Wild cervids‡
	High-risk cervids					Slaughtered cervids			Total	
	Found dead or showing clinical signs	Culled from CWD-confirmed farms	Culled from CWD-linked farms	Total		Routine surveillance	Culled from CWD-linked farms	Total		
Imported elk	6/63 (9.5)	5/279 (1.8)	7/278 (2.5)	18/620 (2.9)		4/167 (2.4)	1/31 (3.2)	5/198 (2.5)	23/818 (2.8)	NA
Initial domestic transmission§	1/22 (4.5)	8/34 (23.5)	8/208 (3.8)	17/264 (6.4)		1/65 (1.5)	1/102 (1.0)	2/167 (1.2)	19/431 (4.4)	NA
Endemic	47/128 (36.7)	276/2,894 (9.5)	62/988 (6.3)	385/4,010 (9.6)		2/126 (1.6)	NA	2/126 (1.6)	387/4,136 (9.4)	0/2,867
Total	54/213 (25.4)	289/3,207 (9.0)	77/1,474 (5.2)	420/4,894 (8.6)		7/358 (2.0)	2/133 (1.5)	9/491 (2.0)	429/5,385 (8.0)	0/2,867

*Values are no. CWD-positive/no. tested (%). CWD, chronic wasting disease; NA, not applicable. †Imported elk stage was 2001–2005; initial domestic transmission was 2010; endemic stage was 2016–2024. ‡CWD surveillance of wild cervids in South Korea began in 2014. The data indicate the number of wild cervids tested during the period 2014−2024. Most (85.4%) tested wild cervids are Korean water deer (*Hydropotes inermis argyropus*), which is the dominant wild cervid species on the mainland of South Korea. On Jeju Island, testing focused on Siberian roe deer (*Capreolus pygargus*), the region’s resident species. §In 2010, 493 cervids culled from 14 farms as part of tuberculosis control measures were also tested for CWD; all were CWD-negative.

**Figure 1:**
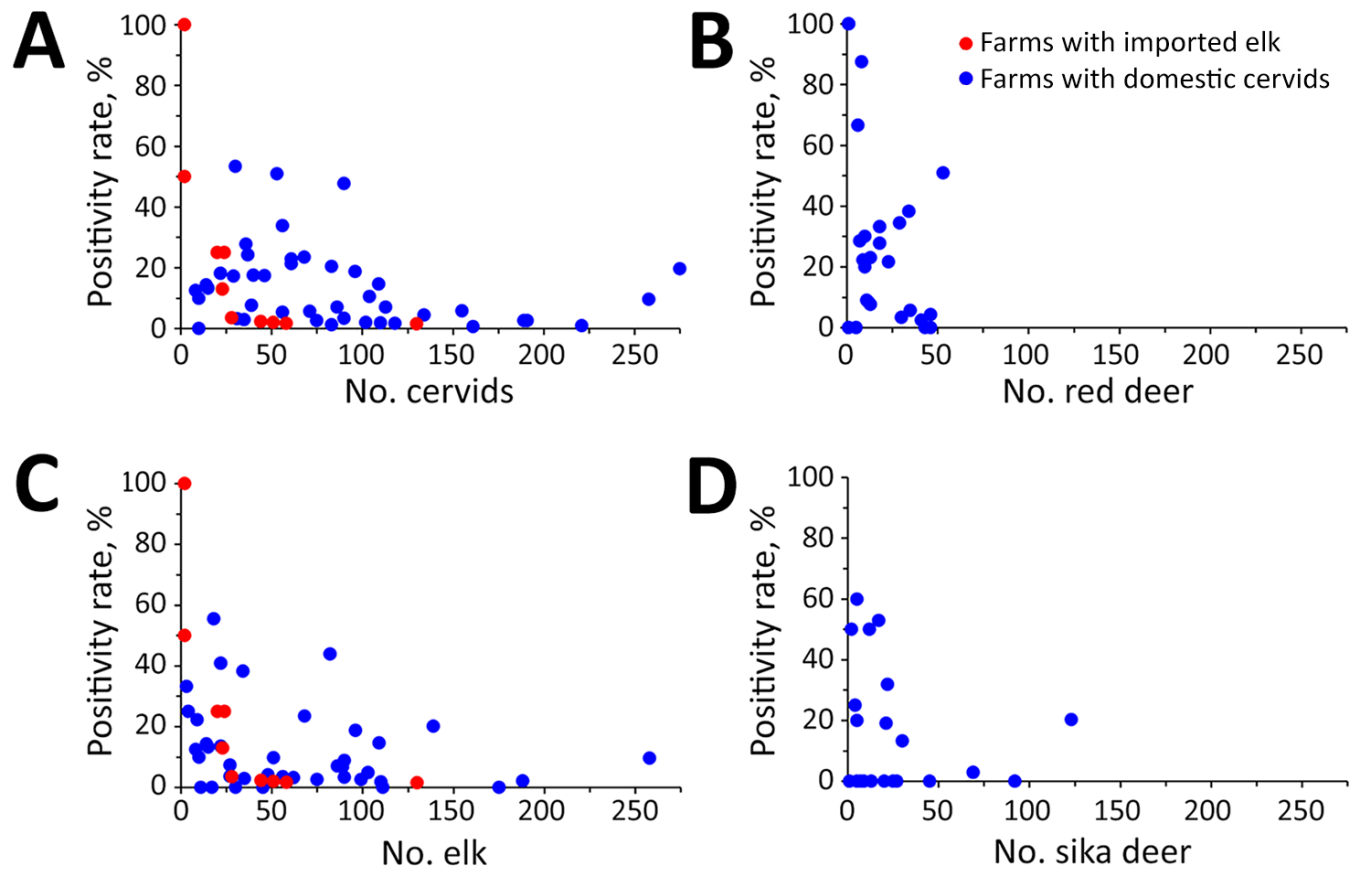
Chronic wasting disease positivity rates in farmed cervids, South Korea, 2001–2024. A) All farmed cervids; B) red deer; C) elk; D) sika deer. Each dot represents a single farm. Positivity rates were calculated as no. animals testing positive/no. animals tested on each farm. Dots reflect the farm’s cervid population because all farmed cervids were culled and tested on farms with positive cases. For species-specific analyses, each cervid species on each farm was assessed separately.

Herd size on the 53 CWD-affected farms varied widely, ranging from 2 to 275 cervids (Figure 1). More than 70% of the farms raised <100 animals; only 3 farms had >200 animals. The number of CWD-positive animals also varied widely across farms; 38 farms had <10 CWD-positive animals, and 11 of those reported only 1 case (Figure 1, panel A). The highest number of CWD-positive animals on a single farm was 54 of 275 animals, corresponding to a positivity rate of 19.6%. Among the farms, 39 (73.9%) had CWD positivity rates <20%, of which 26 (49% of the 53 farms) had rates <10%. In contrast, 4 farms exhibited positivity rates of >50%, 2 of which had only 2 cervids. Analysis by species showed that 42.3% of the farms raising red deer, 52.4% of the farms raising sika deer, and 58.0% of farms raising elk had CWD positivity rates <10% (Figure 1, panels B–D). Positivity rates of >50% were more commonly observed in red and sika deer (19.0%–19.2%) than in elk (6.0%).

**Figure 2:**
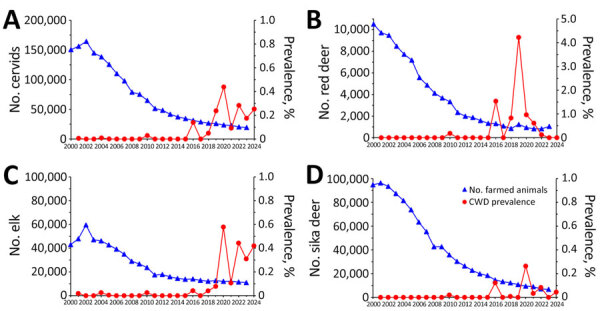
Annual CWD prevalence in farmed cervids, South Korea, 2001–2024. A) All cervids; B) red deer; C) elk; D) sika deer. Prevalence (%) was calculated as no. CWD-positive cervids/nationwide farmed cervid population for each species per year. Population data for 2024 was not available, so 2023 population data was used to calculate prevalence for 2024. CWD, chronic wasting disease.

We investigated the annual nationwide CWD prevalence from its first detection in 2001 through 2024. We calculated prevalence by dividing the number of CWD cases by the total farmed cervid population in the country for each corresponding year. The imported elk stage (2001–2005) and the initial domestic transmission stage (2010) both showed low (<0.3%) CWD prevalence, whereas the endemic stage (2016–2024) exhibited markedly higher and more variable prevalence rates (Figure 2). The highest (4.22%) prevalence occurred in red deer in 2019 (Figure 2, panel B). Although prevalence in elk remained relatively stable during 2022–2024 (Figure 2, panel C), prevalence declined to near zero in red and sika deer (Figure 2, panels B, D). Of note, the farmed cervid population declined markedly over the 2 decades we studied, particularly in red and sika deer. Red deer numbers fell from 9,712 in 2001 to 1,040 in 2023, and sika deer numbers fell from 96,282 to 6,673 over the same period.

During 2001–2024, South Korea confirmed a total of 429 CWD cases across 3 cervid species on 53 farms, most of which occurred during the endemic stage (2016–2024). CWD has progressed from an initial localized cluster to nationwide endemic distribution (Appendix Figure). That geographic expansion could be associated with 2 major factors. One is the residual stock of high-risk imported cervids from Canada that could not be fully traced during the initial control measures of the imported elk stage (*7*). The other is unrestricted movement of cervids between farms, which is difficult to manage because no animal tracking system is available for farmed cervids in South Korea (*10*). In addition, infectious prions can remain in the farm environment, even after intensive decontamination measures, and farms could serve as long-term reservoirs for recurrent CWD (*11*–*14*). 

## Conclusions

Although CWD prevalence in farmed red and sika deer has declined in South Korea, approaching zero in recent years, it remains endemic at low levels in elk, and overall prevalence is ≈0.4%. The sharp contrast between North America’s increasing CWD rates (*12*) and South Korea’s decline in CWD prevalence could be associated with contextual and policy differences. South Korea implemented an aggressive national policy to eliminate CWD, which is a feasible strategy in that country because CWD remains confined to farmed cervids (*10*,*15*). Adherence to the current disease control policy is projected to manage the disease at its current low prevalence of <0.5%. Although the risk for CWD transmission to humans is believed to be low (*12*), South Korea’s continued efforts to reduce CWD prevalence among farmed cervids will limit human exposure and help mitigate such risks.

Appendix Additional information on chronic wasting disease in farmed cervids, South Korea, 2001–2024.
