# Supplementary material for: Chronic Wasting Disease in Farmed Cervids, South Korea, 2001–2024
Source: Emerg Infect Dis. 2026 Apr;32(4):614–8. doi: 10.3201/eid3204.251046 (PMC13094843; doi:10.3201/eid3204.251046)
Supplement: Appendix — Additional information on chronic wasting disease in farmed cervids, South Korea, 2001–2024. [file 25-1046-Techapp-s1.pdf]

Article DOI: https://doi.org/10.3201/eid3204.251046

EID cannot ensure accessibility for supplementary materials supplied by authors. Readers who have difficulty accessing supplementary content should contact the authors for assistance.

Chronic Wasting Disease in Farmed Cervids, South Korea, 2001–2024

Appendix

Supplementary Methods

The following CWD control and eradication measures have been implemented in South Korea since 2001 (detailed elsewhere [*1*]). The only exception is PMCA analysis on soil samples, which was introduced in 2016 (*2*,*3*).

CWD surveillance in farmed cervids

Upon observation of clinical signs consistent with CWD in farmed cervids (e.g., progressive weight loss, ataxia/lack of coordination, hypersalivation, polydipsia, and polyuria) or finding dead animals, farm owners were required to notify local veterinary authorities. Local veterinary personnel then collected tissue samples (the obex and retropharyngeal lymph nodes), and submitted them to the Animal and Plant Quarantine Agency (APQA) for CWD diagnostic testing. Mandatory CWD testing was also implemented for all cervids consigned to slaughterhouses (although the use of such facilities is not mandatory for all cervid slaughter procedures), with samples collected directly at the slaughter facility.

CWD surveillance in wild cervids

Surveillance for CWD in wild cervids was preferentially conducted within regions where CWD-affected farms were located. The sample size for this surveillance was statistically determined to detect CWD with 95% confidence, assuming a 1% infection rate in the wild population within these affected regions. Tissue samples were collected by the Wildlife Management and Rescue Centers from animals found injured or dead within these targeted areas, and submitted to the APQA for CWD diagnostic testing.

CWD diagnostics

CWD diagnosis in cervid populations was performed at the WOAH Reference Laboratory for CWD, which operates out of the APQA in South Korea. The testing protocol involved the examination of obex and retropharyngeal lymph node specimens, utilizing the IDEXX HerdChek BSE-Scrapie Antigen Test. CWD-positive cases identified by the primary test were confirmed using the TeSeE Western Blot (Bio-Rad) assay.

Depopulation

Once a CWD diagnosis was confirmed by APQA, immediate and thorough depopulation of the affected farm was initiated. All remaining cervid animals on the positive farm were immediately culled and tested for CWD using the methods described above. In addition, a tracing investigation was immediately initiated; all cervids on other farms that had either received animals from or sent animals to the CWD-positive farm within the preceding five years were also culled and tested for CWD.

Environmental decontamination and restocking

Following herd depopulation on the positive premises, extensive environmental decontamination was performed: the upper layer of soil was removed to a depth of 30 cm in high-risk areas (around barns, feeding troughs, and manure storage sites) and to a depth of 5 cm in all other areas of the farm. Simultaneously, all incinerable materials within the affected farms, such as bedding and leftover feed, were incinerated. Subsequently, the removed topsoil, incineration ash, and animal carcasses were buried on-site at the respective farms after thorough application of a 2N NaOH solution, and the burial sites were subsequently secured from other farm areas by the construction of a metal fence. Finally, the soil and other environmental surfaces across the entire farm were treated with 2N NaOH a minimum of three times, with an interval of 2 to 3 weeks between each application. After the chemical treatments, if the farm owners intended to restock cervids, soil samples were collected and examined using protein misfolding cyclic amplification (PMCA) technique as described in detail (*2*). To this end, we collected two sets of soil samples with 1-2 month intervals between collection. If PMCA seeding activity was identified in either sample set from a particular farm, the farm was subjected to three more rounds of 2N NaOH treatment at intervals of 2-3 weeks, followed by another PMCA screening utilizing two new sample sets collected 1-2 months apart. Restocking of cervid animals was permitted only after PMCA results confirmed the complete absence of prion seeding activity on the remediated site.

References

1. Park KJ, Park H-C, Lee Y-R, Mitchell G, Choi YP, Sohn H-J. Detection of chronic wasting disease prions in the farm soil of the Republic of Korea. MSphere. 2025;10:e0086624. PubMed https://doi.org/10.1128/msphere.00866-24

2. Park KJ, Park HC, Lee YR, Roh IS, Mitchell G, Choi YP, et al. Addressing chronic wasting disease in Korean farms: topsoil removal and 2N NaOH treatment before cervid restocking. Prion. 2025;19:20–7. PubMed https://doi.org/10.1080/19336896.2025.2527588

3. Sohn HJ, Park KJ, Roh IS, Kim HJ, Park HC, Kang HE. Sodium hydroxide treatment effectively inhibits PrP^{CWD} replication in farm soil. Prion. 2019;13:137–40. PubMed https://doi.org/10.1080/19336896.2019.1617623

Appendix Table 1. Yearly CWD Surveillance data by category in South Korea, 2001-2024*

Year	Farmed cervids				Wild cervids
	High-risk animals	Slaughtered animals	Others†	Total farmed cervids	
**2001**	9/186	-	-	9/186	-
2002	0/109	-	-	0/109	-
2003	0/2	-	-	0/2	-
**2004**	7/61	5/40	-	12/101	-
**2005**	2/262	0/158	-	2/420	-
2006	0/16	0/199	-	0/215	-
2007	0/86	0/116	-	0/202	-
2008	0/76	0/58	-	0/134	-
2009	0/186	0/67	-	0/253	-
**2010**	17/264	2/167	0/493	19/924	-
2011	0/249	0/79	0/54	0/382	-
2012	0/15	0/31	0/83	0/129	-
2013	0/72	0/97	-	0/169	-
2014	0/2	0/33	0/11	0/46	0/249
2015	0/0	0/52	-	0/52	0/216
**2016**	44/359	0/34	-	44/393	0/113
2017	0/5	0/13	-	0/18	0/82
**2018**	11/354	2/16	-	13/370	0/185
**2019**	62/394	0/11	-	62/405	0/376
**2020**	104/673	0/7	-	104/680	0/332
**2021**	21/421	0/3	-	21/424	0/332
**2022**	60/554	0/11	-	60/565	0/342
**2023**	34/637	0/18	-	34/655	0/306
**2024**	49/613	0/13	-	49/626	0/334
Total	420/5,596	9/1,223	0/641	429/7,460	0/2,867

*Bold font indicates years in which CWD occurred. CWD, chronic wasting disease.
†This category represents cervids that were culled in association with tuberculosis control measures and subsequently tested for CWD in addition to tuberculosis. All tested animals in this category were negative for CWD.

Appendix Table 2. Farmed cervid population in South Korea*

Region	Red deer	Elk	Sika deer	Reindeer	Other species	Total
Gangwon	66	468	1,111	23	53	1,721
Gyeonggi	31	930	1,178	7	33	2,179
Chungnam	116	4,052	497	-	49	4,714
Chungbuk	184	1775	273	4	-	2,236
Jeonnam	72	1,898	544	-	-	2,514
Jeonbuk	168	562	733	9	48	1,520
Gyoungnam	308	441	1,422	-	27	2,198
Gyoungbuk	13	575	273	141	109	1,111
Jeju	29	101	179	-	20	329
Seoul	-	-	-	-	-	-
Busan	3	2	41	-	11	57
Daegu	50	56	133	-	1	240
Incheon	-	4	106	-	-	110
Gwangju	-	-	-	-	110	110
Daejon	-	-	-	-	-	-
Ulsan	-	78	256	-	-	334
Sejong	-	38	17	-	-	55
Total	1,040	10,980	6,763	184	461	19,428

*The farmed cervid population data in this table represents the status as of 2023. Data for 2024 is not yet available. The data were obtained from reports published by the Ministry of Agriculture, Food and Rural Affairs, which are available at https://www.mafra.go.kr/bbs/home/795/572197/artclView.do.

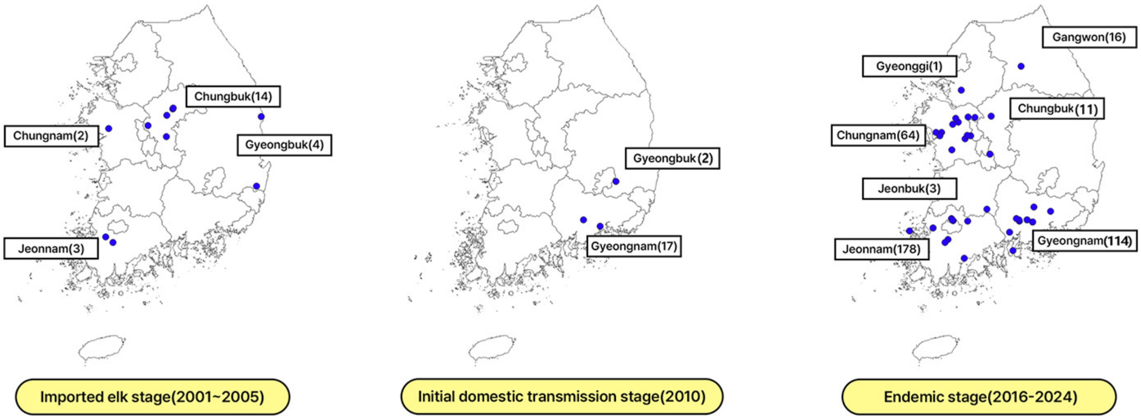

Appendix Figure. Distribution of CWD by stage in South Korea, 2001–2024. This figure illustrates the geographic distribution of CWD-confirmed farms across three distinct epidemiologic stages in South Korea. The stages are as follows: Imported elk stage (2001–2005), Initial domestic transmission stage (2010), and Endemic stage (2016–2024). Blue dots represent the geographic locations of individual CWD-confirmed farms. Labels indicate the province name and the cumulative number of CWD-infected cervids in that province during the respective stage.
